# Sustainability in Participatory Digital Health: Identifying Key Influencing Factors Across Contexts

**DOI:** 10.1055/s-0046-1816591

**Published:** 2026-11-03

**Authors:** Kerstin Denecke, Octavio Rivera-Romero, Jeppe Eriksen, Elia Gabarron, Rebecca Grainger, Fernando Martin-Sanchez, Iris ThieleIsip Tan, Talya Miron-Shatz, Rolf Wynn, Carolyn Petersen

**Affiliations:** 1 Bern University of Applied SciencesBernSwitzerland; 2 Department of Electronic Technology, Universidad de SevillaSevillaSpain; 3 Department of Sustainability and Planning, Aalborg UniversityDenmark; 4 Department of Education, ICT and Learning, Østfold University CollegeHaldenNorway; 5 University of Otago WellingtonWellingtonNew Zealand; 6 Fernando Martin-Sanchez, Medical Informatics Department, Hospital Universitario “LaPaz”MadridSpain; 7 Medical Informatics Unit, College of Medicine, University of the PhilippinesManilaPhilippines; 8 Ono Academic CollegeKiryat OnoIsrael; 9 Department of Clinical Medicine, UiT The Arctic University of NorwayTromsøNorway; 10 Carolyn Petersen, Department of Artificial Intelligence and Informatics, Mayo ClinicRochesterMinnesotaUnited States

**Keywords:** Participatory Health Informatics, Sustainability, Equity, Trust, Digital Health, Patient Engagement

## Abstract

**Background:**

Information and communication technology including Apps and tools based on artificial intelligence present new opportunities and challenges for the achievement of sustainability.

**Objective:**

We aimed to identify factors influencing sustainability in participatory digital health solutions to advance the understanding of sustainability as applied to participatory digital health solutions, including current prioritisation and risks.

**Methods:**

We collected from the literature factors relevant to sustainability in digital health and evaluated the relevance of these factors in five real-world participatory digital health case studies taken from the literature.

**Results:**

We identified five groups of factors: individual, technological context, social norms, data, and digital health solutions factors. Individual and technological context factors were considered to have the highest impact in the five case studies, while social norms, data, and algorithms showed variability. Key findings include the importance of digital literacy and robust technology access, as seen in telemedicine during climate emergencies, and challenges such as low digital self-efficacy and algorithmic bias. Our results underscore the need for improved data integration, transparency, and algorithmic fairness, emphasizing tailored, context-specific strategies to enhance adoption, scalability, and alignment with sustainability goals.

**Conclusion:**

Our findings suggest that for achievement of sustainability in the participatory digital health ecosystem, individual and context factors generally are considered more important than factors relating to social norms, and those relating to data and algorithms.

## 1. Introduction

While digital health solutions are now widely implemented to address the challenges of population health and healthcare delivery, the sustainability of these technology approaches is uncertain. Recent discussions have offered definitions of sustainable digital health, the common theme being that sustainability is multifaceted [ 1 , 2 ]. A clear and operationalised definition of sustainability in digital health remains elusive, so there is an urgent need to address this barrier to systematic and expected embedding of sustainability in digital health solutions. To address this vexing and pressing issue, as a group of international experts in participatory digital health, we have collated from the literature factors relevant to sustainability in digital health and evaluated the relevance of these factors in five real-world participatory digital health case studies taken from the literature.

The digital health community is increasing considering sustainability and attempting to define what this looks like in practice [ 1 , 3 , 4 ]. We have found the recent definition from Hammad *et al.* , which proposes that digital health is “ *the ethical, equitable, and environmentally responsible use of digital technologies to improve health outcomes, enhance healthcare delivery, and promote well-being while ensuring long-term viability and minimizing negative impacts on society and the environment* ” [ 1 ]. This definition centres the principles of ethical use of digital health solutions including privacy, security, consent, and transparency in data use. Furthermore, the expectation is that digital health solutions should be accessible to all, whether this is applied to a community or in both high- and low-income countries. The final foundation in the definition is the environmentally responsible use of digital health, which can mean working to reduce the environmental footprint or minimize energy use and all forms of waste. The importance of avoiding negative impacts on society and environment is rearticulated in the final clause of the definition. As with all health interventions, the goal is to enhance health outcomes and healthcare by offering timely and effective solutions while promoting well-being. An important and sometimes overlooked aspect of sustainability articulated in the definition is the need to ensure long-term viability from the outset, implying that digital health solutions should be designed to be maintained as long as they remain useful and financially advantageous. The definition provides a launch point for further consideration of the aspects of sustainable digital health. Digital health must however be considered in the wider context of sustainability initiatives.

A major driver for embedding sustainability in all human activities has been the United Nations’ (UN) 2030 Agenda for Sustainable Development 1 , adopted by all member states in 2015. The 17 Sustainable Development Goals (SDGs) aim to address current global challenges of poverty, inequitable health outcomes and access to education, and discrimination and social exclusion while increasing economic growth and managing climate change and preserving nature [ 5 ]. The World Health Organisation’s (WHO’s) strategy on digital health (WHO, 2021 [ 6 ]) emphasises that information and communication technology including apps and tools based on artificial intelligence presents new opportunities and challenges for the achievement of all 17 SDG. There is a growing consensus in the global health community that the strategic and innovative use of digital and cutting-edge information and communications technologies will be an essential enabler of the WHO’s aspirations for global improved health [ 6 ]. It seems essential that further consideration of digital health solution sustainability must be underpinned by the UN’s SDGs.

The focus of this paper will be participatory digital health solutions, which can be defined as information technology that delivers tools with a focus on individual-centred care which may address individuals in the role of patients but also the health of society as a whole (adapted from [ 7 ]). We seek to identify factors influencing sustainability in participatory digital health solutions to advance the understanding of sustainability as applied to participatory digital health solutions, including prioritisation and risks.

## 2. Methodology

Five case studies of participatory digital health solutions that address some of the sustainable development challenges defined by the United Nations have been identified in the literature. These case studies were used to assess the relevance of specific factors that may impact the sustainability of a digital health solution. In the following work, we describe the process of selecting the case studies, the collection of relevant factors, and the scoring of factors per case study.

### 2.1.Selection of Case Studies

To select case studies, we reviewed the UN SDGs and selected goals that are of relevance for participatory health solutions. Specifically, we considered the five sustainable development goals:

- Goal 3: Ensure healthy lives and promote well-being for all at all ages;
- Goal 5: Achieve gender equality and empower all women and girls;
- Goal 10: Reduce inequality within and among countries;
- Goal 12: Ensure sustainable consumption and production patterns;
- Goal 13: Take urgent action to combat climate change and its impacts.

Since literature comparing efforts between countries is largely absent and our focus was to develop a preliminary framework for sustainability of participatory digital health solutions, we did not include SDG 10. Then, we used keywords found within the statements of four goals that address at least one of these goals. The selected case studies are summarized in Section 3.1.

### 2.2. Selection of factors impacting sustainability digital health solutions

To identify factors that affect sustainability of participatory digital health solutions, we first identified frameworks to underpin design of sustainable digital health solutions proposed in the literature. To select frameworks, we searched for papers matching keywords related to model, sustainability and health:

- Models: Framework OR Model OR Guidelines OR standard OR best practice OR policy
- Sustainability: Sustainab* OR ethic* OR Equit* OR responsib* OR “Digital Literacy” OR “self-efficacy” OR “Technology access” OR Fair* OR “engagement” OR “acceptance” OR “adoption” OR “inclusive”
- Health field: vv“digital health” OR “mobile health” OR “health informatics” OR “medical informatics” OR “mhealth” OR “connected health” OR “telehealth” OR “telemedicine” OR “digital intervention” OR “digital health intervention”

We identified seven frameworks covering the dimensions digital health equity, technology acceptance, trust, and ethics. Specifically, we used:

- Digital health equity: Framework for digital equity [ 8 ] and Mobile health ecosystem guidelines [ 9 ];
- Technology acceptance: Technology Acceptance Model for Resource-Limited Settings [ 10 ] and UTAUT2 [ 11 ];
- Trust: Digital responsibility goals [ 12 ];
- Ethics: Guidelines for ethical planning, implementation and evaluation of mHealth for treatment adherence [ 13 ] and Universal principles of ethics in research with human subjects [ 14 – 16 ].

From the identified frameworks, one author (ORR) extracted all factors suggested by any of the frameworks ( Table 1 ). The factors were grouped into five categories: individual, technological, social norms, data, and algorithms. We set up an online survey that briefly described each of the five case studies and listed all 34 factors. All 10 co-authors were asked to score the impact of each factor on sustainability for each single case study on a 5-item scale based on their knowledge of and experience in participatory health. The co-authors are all members of the IMIA Social Media Group Participatory Health and Social Media. The aim was to describe which factors these experts considered to impact on the sustainability of digital health solutions in the case studies considered.

**Table 1:** Factors to be rated by the survey participants for each case example.

Category of factors	Factors
A. Individual factors: Factors related to the individual might affect the use of the proposed solution.	Digital literacy, Digital self-efficacy, Attitude towards use, Hedonic motivation, Age, Gender, Experience, Behavioural intention, Habit.
B. Technological context factors: Factors related to the technological context might affect the use of the proposed solution.	Technology access, Interdependence, Implicit tech bias, Community infrastructure, Healthcare infrastructure, Tech policy.
C. Social norms factors: Factors related to the social norms and support might affect the use of the proposed solution.	Social norms and ideologies, Community tech norms, Patient-Tech-Clinician relationship, Community partners, Incentives.
D. Data factors: Factors related to the data (collection, storage, transmissions, etc.) might affect the use of the proposed solution.	Data standards, Confidentiality, Privacy, Security, Data fairness, Transparency, Accuracy
E. Digital health solution factors: Factors related to the digital health solution, e.g. algorithms implemented in the solution might affect its use.	Algorithmic bias, Trustworthy algorithms, Perceived ease of use, Perceived usefulness, Costs, Human agency and identity, Inclusivity, Adaptive technology.

To facilitate analysis of the results, we transformed the original verbal ratings into numerical values. The ratings were converted as follows:

- Very high extent” was assigned a value of 5;
- High extent” was assigned a value of 4;
- Neither high nor small extent” was assigned a value of 3;
- Small extent” was assigned a value of 2;
- Very small extent” was assigned a value of 1.

## 3. Results

### 3.1. Case studies

Table 2 describes the five identified case studies.

**Table 2:** Summary of representative case studies, SDG – Sustainable Development Goals.

Case study	Brief description and relevant SDG goals
Case study 1: App providing free accurate sexual health information [ 17 ] Context: Women in Western and Central African countries	A usable, accessible app providing culturally appropriate content in several languages was designed using a participatory process involving experts and potential users. The final version achieved the inclusivity, i.e., ‘all at all ages’. SDGs: 3, 5
Case study 2: SMS-based digital tool to support HIV care [ 18 ] Context: People with HIV in Rwanda during the COVID-19 pandemic	WelTel, an SMS-based digital tool developed to support HIV care in Rwanda, provided daily monitoring and support for people and their contacts through COVID-19 isolation and quarantine. Stakeholders credited WelTel with improving access and adherence to infection prevention and control measures, aiding understanding of transmission, and informing public health decision makers about home-based care. SDGs: 12
Case study 3: Antimicrobial resistance in farming [ 19 ] Context: Precision livestock farming incorporating digital data collection	Prevention of antimicrobial resistance (AMR) is critical because rising temperatures associated with climate change creates a more positive environment for disease-causing pathogens. A precision livestock farming approach that incorporates digital data collection into livestock production practices was developed to support early identification of potentially ill animals for examination and quarantine. Reducing disease spread and antimicrobial use slows development of AMR. SGDs: 12
Case study 4: System supporting generation and ongoing monitoring of climate change and health-related data [ 20 ] Context: Low- and middle-income countries (Africa and Asia)	Health and demographic surveillance sites (HDSSs) in low- and middle-income countries in Africa and Asia may have an established infrastructure with longitudinal population cohort data, but often lack climate-health specific data. The Change and Health Evaluation and Response System supports generation and ongoing monitoring of climate change and health-related data within HDSSs via a database to analyze health and environmental exposures at the individual, household, and community levels. SGDs: 13
Case study 5: Telemedicine in climate-related events [ 21 ] Context: Before, during, and after climate-related events	Lack of access to care through established care team relationships and clinics is a major problem immediately before, during, and after climate-related events. Evacuation shelters are often set up in nonmedical facilities (e.g., school gymnasiums) that lack medical equipment and examination space. After a 2018 hurricane in the southeastern United States a telemedicine service proved to be an effective in-shelter alternative to Emergency Medical Service and hospital Emergency Department care. SGDs: 13

### 3.2. Survey Participants

The survey included 10 participants, of whom 60% (6 out of 10) were female. The participants represented a wide range of nationalities, being from eight different countries: Norway (2), Spain (2), Switzerland, New Zealand, Philippines, United States, Israel and Denmark. In terms of professional roles, 60% of participants (6/10) worked exclusively in academia, while 10% (1/10) worked in public health. In addition, 20% (2/10) worked in both academia and public health, and 10% (1/10) worked in both academia and the private health sector. Participants’ educational backgrounds were diverse, including psychology (2 participants), other health sciences (2), medicine (3), health informatics (4), physiotherapy (1), computer science or engineering (2), psychiatry (1) and sociology (1).

Participants also had extensive experience in participatory health informatics. While 20% (2 out of 10) had 5-10 years of experience in the field, the majority, 80% (8 participants), reported more than 10 years of experience. v

### 3.3. Survey Analysis Results

The relative importance of the five domains across all use cases is summarized in Figure 1 . The complete survey results is delivered as Supplementary Material.

**Figure 1.**
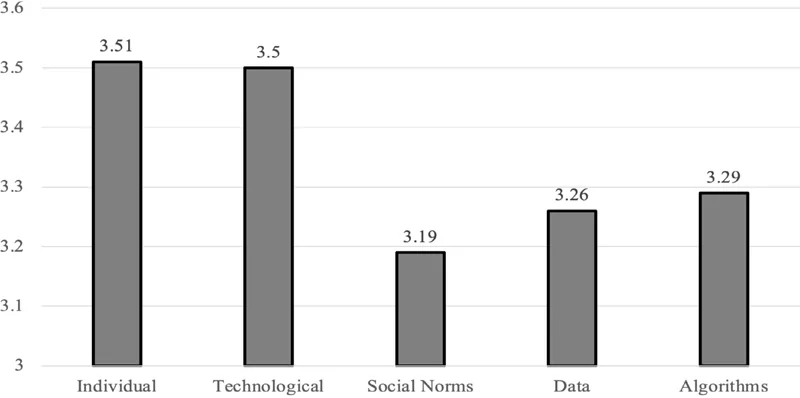
Factors to be rated by the survey participants for each case example.

Factors were to be scored with a value between 1 and 5. Overall, individual and technological context factors were rated as the most influential, with average scores of 3.51 and 3.50, respectively. In contrast, social norms factors were perceived as the least impactful (average score: 3.19), highlighting potential cultural and societal barriers to the adoption of these solutions. The results show significant contrasts in scores across the factors evaluated. The highest score observed was 4.9, recorded in Case 5 for “Technology Access”, highlighting the critical importance of a robust technological infrastructure to support effective telemedicine in climate-related emergencies. Conversely, the lowest score was 1.8 identified in Case 2 for “Digital Self-Efficacy”, highlighting significant challenges in user readiness and confidence to use SMS-based tools in HIV care. In the following, we study in more detail the results from the 5 different categories of factors.

#### Individual Factors

This group of 9 factors encompasses aspects such as digital literacy, behavioral intention, and habit. The average values for all factors of this category were between 2.52 and 4.08. The factor “Attitude towards use” showed the highest average score (4.08), with a score of 4.2 for Case 2 and Case 4 demonstrating the importance of user acceptance for sustainable digital health solutions. Hedonic motivation scored low across all cases, with a minimum of 2.1 in Case 3, indicating limited importance of fun or pleasure in adoption around the topics the cases covered. The second highest score has the factor Behavioral Intention with an average of 3.86; Case 4 achieved a notable 4.2 in average for this factor, reflecting strong user intentions to adopt digital health tools.

#### Technological Context Factors

This category comprises factors related to technology access and community or healthcare infrastructure as they are critical enablers of sustainability. The values of the 6 factors range between 2.94 and 4.34. The highest average values are achieved for the two factors Technology Access and Community infrastructure. The factor Technology Access achieved the highest score of the survey for one case (4.9 for Case 5). This result highlights the reliance on robust infrastructure in digital health solutions. The factor Community Infrastructure scored 4.6 for Case 4, showcasing the importance of cellular and community resources for successful implementation. The lowest average score in this category (2.94) was achieved by the factor Implicit Tech Bias.

#### Social Norms Factors

The 5 social norms factors include societal and community influences on technology adoption. The average scores were between 2.54 and 3.5. For Case 1 and Case 2, the factor Social Norms and Ideologies scored high with an average of 4.0, indicating strong alignment with cultural beliefs in sexual health and HIV care. The lowest scores were assigned to the factor Patient-Tech-Clinician relationship. On average, this factor scored 2.54. For Case 3, only an average value of 1.8 was achieved.

#### Data Factors

Data factors comprise 7 factors related to the management, storage, and governance of data. Average scores range from 2.48 to 3.72. The factor Data Standards achieve the lowest average score (2.48), with the lowest score of 1.9 in Case 1. The factor Privacy received the highest score with 3.72. For Case 2, the score was the highest with 4.5. The factors Accuracy and Confidentiality scored with values of 3.52 and 3.48, respectively. These judgments show the importance of secure and accurate data handling and storing.

#### Digital Health Solutions Factors

The digital health solution factors include Algorithmic bias, Trustworthy algorithms, and Inclusivity. Algorithmic bias scored lowest with 2.44, while the factor Perceived Usefulness was judged as most important with an average score of 4.3. For Case 2, the factor Algorithmic Bias scored only 1.8, pointing to challenges in fairness for SMS-based systems. Perceived Usefulness achieved a score of 4.5 for Case 4, demonstrating the role of effective algorithms in meeting user needs. Another quite highly ranked factor in this category was Costs, which had an average value of 4. For Case 1 and Case 3 this factor achieved average values of 4.2 and 4.4, respectively.

## 4. Discussion

### 4.1. Summary of Findings

This manuscript highlights areas to be considered in the sustainable development and implementation of sustainable participatory digital health solutions, categorized into individual, technological context, social norms, data, and digital health solutions factors. Individual and technological context factors were considered to have the highest impact, while social norms, data, and algorithms showed variability. Key findings include the importance of digital literacy and robust technology access, as seen in telemedicine during climate emergencies, and challenges such as low digital self-efficacy and algorithmic bias. Our results underscore the need for improved data integration, transparency, and algorithmic fairness, emphasizing tailored, context-specific strategies to enhance adoption, scalability, and alignment with sustainability goals.

### 4.2. Impact of Individual and Technological Context Factors on the Sustainability of Participatory Digital Health Solutions

The integration of participatory digital health solutions into healthcare has the potential to provide several positive effects, such as prevention [ 19 ], health promotion [ 17 ], care support [ 18 , 21 ], and surveillance [ 20 ]. It is crucial to ensure that these benefits also contribute to sustainability.

Based on the expert views, individual and technological context factors emerged as critical determinants influencing the adoption and sustainability of these solutions. Individual factors, particularly digital literacy and behavioral intentions to adopt tools in climate-health monitoring, were scored high among the survey respondents. Digital literacy has been described in the literature as playing a significant role in the sustainability of digital health solutions [ 22 – 24 ]. Individuals with high digital literacy could be more likely to use digital technologies in ways that minimize the need for physical visits to healthcare facilities [ 25 ], thereby significantly lowering carbon emissions associated with patient transportation, a major contributor to healthcare-related environmental impact. Research in other fields has shown that by increasing digital literacy, individuals are additionally more likely to choose green technologies [ 26 ]. This may also be applicable to the healthcare field. Strategies to increase digital literacy could empower users to adopt environmentally friendly digital tools, while advancements in technology design could reduce energy consumption and electronic waste [ 22 , 27 ].

Technological context factors, such as technology access and community infrastructure, emerged in our survey as relevant enablers of the sustainability of participatory digital health solutions. Guaranteeing a good access to technology, particularly in telemedicine during climate-related emergencies, highlights the importance of robust digital infrastructure [ 21 ]. This infrastructure could facilitate remote healthcare delivery, and thereby reduce the reliance on energy-intensive hospital operations and travel. However, the ecological benefits also depend on the energy sources powering the technological infrastructure. There is a need to transition to renewable energy sources for data centers, telecommunication networks, and end-user devices to maximize ecological gains. Another technological context factor highlighted by the survey respondents for their role on sustainability was the impact of community infrastructure. This was scored highly in the climate-health monitoring use case [ 20 ], further emphasizing the potential of leveraging localized resources to reduce ecological footprints. By integrating digital solutions with existing community resources, such as solar-powered cellular networks [ 28 ], participatory digital health solutions can align with global efforts to combat climate change.

### 4.3. Impact of Social Norms Factors on the Sustainability of Participatory Digital Health Solutions

While in the survey social norms factors were found to be the least impactful on average for sustainability of participatory digital health solutions across the five case studies, these factors scored highest in the first two case studies on sexual health and HIV. In a randomized controlled trial on the use of a mobile app for the delivery of sexual and reproductive health (SRH) for female adolescents in the United States [ 29 ], use of the app was associated with improved confidence in seeking SRH care at a clinic and that users can choose to use contraception when they have sex. In-depth interviews with women in Bangladesh have shown that SRH information provided on mobile devices has the potential to empower women in highly patriarchal societies who face social and religious taboos related to seeking healthcare [ 30 ]. A randomized controlled trial among sexually active university students in Uganda demonstrated that a mobile app improved SRH knowledge scores, access to contraception, and HIV voluntary testing and counseling [ 31 ]. The mobile app intervention was considered viable given that Ugandan youth also had high access to mobile internet.

Among the social norms factors, patient-tech-clinician relationships consistently averaged low in the survey across the five case studies for impact on sustainability of participatory digital health solutions. The average score was lowest for the case study related to antimicrobial resistance (AMR) and digital data collection for precision farming, perhaps a reflection of knowledge gaps regarding AMR. In a study evaluating knowledge on AMR of physicians and pharmacists [ 32 ], the majority of physicians believed that not addressing the nidus of infection was most contributory to AMR, in contrast to pharmacists who cited the disproportionate use of antibiotics in livestock.

### 4.4. Impact of Data and Digital Health Solutions Factors on the Sustainability of Participatory Digital Health Solutions

Across the five case studies, data standards garnered low average survey scores for its impact on the sustainability of participatory digital health solutions. Perhaps this reflects the elusive goal of interoperability and uneven implementation of Fast Healthcare Interoperability Resources (FHIR), which may be due to the availability of multiple versions, safety and legal considerations, and the requirement for a FHIR server [ 33 ].

For digital health solutions factors, the lowest scoring factors in the survey were algorithmic bias and trustworthy algorithms. The lowest average scores for algorithmic bias were for the first two case studies, as these digital health interventions catered to women and persons with HIV mitigating the bias against these groups by representation. Other ways to mitigate algorithmic bias include consumer and patient engagement at the design and development stages, and equitable research methodologies [ 34 , 35 ].

### 4.5. Sustainability and Participation

Besides the findings, this work also presents several learnings related to sustainability. Hameed *et al.* [ 1 ] perceive sustainability as “ethical, equitable, and environmentally responsible use of digital technologies”. In a research context it is often the 17 SDGs introduced by the UN that functions as the frame of reference, and working in the health domain it is especially SDG 3 Good Health and well-being that is of interest. In other words, the concept of sustainability is very broad and multidimensional, especially in an interdisciplinary field such as digital health. Consequently, findings and discussions on this subject help to clarify the type of sustainability that is at hand. This is required if we are to avoid the trap of conceptual stretching [ 36 ], where sustainability means anything and therefore nothing. Conceptual stretching is a known challenge in environmental health, as explained in the editorial by Tee L. Guidotti: “In science, we concentrate on the literal meaning of words and try to the maximum extent possible to remove connotation, ambiguity, and shadings of meaning. We fail!” [ 37 ].

Results from this work show how individual and technological context factors are essential when implementing digital health solutions. The importance of adequate technical infrastructures is recognized widely; however, that the infrastructures have no value if not used by the individuals is not always appreciated. Hence, the development of digital solutions not tailored to those who are most in need of healthcare is a continuous challenge that needs to be addressed when designing and implementing new technologies [ 38 ]. Thus, inclusion and participation in design and implementation processes are favourable both normatively and concretely for the digital healthcare system. Sandman *et al.* (2017), explain this point by distinguishing between instrumental (i.e., practical, outcome-oriented justifications) and substantive rationales (i.e., ethical, rights-based justifications) for patient participation. Based on instrumental rationalities, inclusion of patients is preferable as it improves the relevance of healthcare goals, enhances adherence, and might empower the engaged patients, which in this framework is referred to as capacity building. From the substantive rationality position, patient participation should be enacted as it is most fair to do so, it legitimizes decisions, and it is a way to promote equity in healthcare [ 39 ].

Empirical evidence on Nordic citizens’ experiences, attitudes, and perceptions of the digital healthcare systems shows that the majority of citizens agree that digital healthcare systems have improved accessibility, that their health data is easy to access and understand, and that most are willing to share their digital health data. However, 10 to 20% of the respondents are either unable or uninterested in using the digital healthcare system at all and find it less beneficial [ 40 ]. This perspective is pivotal to keep in mind if we are to develop digital healthcare systems that are genuinely sustainable.

### 4.6. General Observations and Implications for Sustainability

The results show that while individual and technological context factors consistently scored high, significant variability exists in social norms, data, and digital health solutions factors. Strengthening these areas is crucial for achieving the long-term sustainability of participatory digital health solutions. Addressing gaps in user education, infrastructure, data governance, and algorithmic fairness is essential for aligning digital health solutions with the United Nations’ SDGs. Tailored, context-specific strategies will enable better adoption and scalability.

## 5. Conclusion

Sustainability is emerging as an important factor in all facets of life. The WHO has drawn attention to the importance of considering perspectives relating to ethics, equitability, and the environment within healthcare and digital health. In this study, we have determined which factors an expert panel considered most central to the development of sustainable digital health, basing the discussion on five actual cases of implemented digital health solutions. This work highlights the critical role of individual and technological contextual factors in the sustainable implementation of participatory digital health solutions. Digital literacy and robust technology access are paramount to maximising the environmental and health benefits of these solutions. The findings highlight the need for tailored strategies that address the specific needs of diverse populations to ensure that digital health tools are both accessible and effective.

In addition, the variability observed in social norms, data, and drivers of digital health solutions highlights areas that require further attention. Improving data integration, transparency, and algorithmic fairness is essential to build trust and encourage widespread adoption. The study also highlights the importance of transitioning to renewable energy sources to support the technology infrastructure, thereby aligning digital health initiatives with global sustainability goals.

Ultimately, the long-term sustainability of participatory digital health solutions will depend on a holistic approach that integrates individual, technological, social, and data-related factors. By fostering inclusive and equitable digital health ecosystems, we can contribute to the broader goals of sustainable development and improved health outcomes for all.

## Footnotes

1
https://sdgs.un.org/2030agenda
